# Quantitative Susceptibility Mapping and Vessel Wall Imaging as Screening Tools to Detect Microbleed in Sentinel Headache

**DOI:** 10.3390/jcm9040979

**Published:** 2020-04-01

**Authors:** Daizo Ishii, Daichi Nakagawa, Mario Zanaty, Jorge A. Roa, Sami Al Kasab, Amir Shaban, Joseph S. Hudson, Carlos Osorno-Cruz, Stefano Byer, Lauren Allan, James C. Torner, Issam A. Awad, Timothy J. Carroll, Edgar A. Samaniego, David M. Hasan

**Affiliations:** 1 Department of Neurosurgery, University of Iowa Hospitals and Clinics, Iowa City, IA 52242, USA; daizo-ishii@uiowa.edu (D.I.); mario-zanaty@uiowa.edu (M.Z.); jorge-roa@uiowa.edu (J.A.R.); hudsonjs@upmc.edu (J.S.H.); carlos-osorno-cruz@uiowa.edu (C.O.-C.); s614b751@kumc.edu (S.B.); 2 Department of Neurosurgery, University of Tokyo, Tokyo 113 8654, Japan; dnakagawa-tky@umin.ac.jp; 3 Department of Neurology, University of Iowa Hospitals and Clinics, Iowa City, IA 52242, USA; alkasab@musc.edu (S.A.K.); awshaban@gmail.com (A.S.); edgar-samaniego@uiowa.edu (E.A.S.); 4 Department of Surgery, University of Iowa Hospitals and Clinics, Iowa City, IA 52242, USA; lauren-allan@uiowa.edu; 5 Department of Epidemiology, University of Iowa Hospitals and Clinics, Iowa City, IA 52242, USA; james-torner@uiowa.edu; 6 Section of Neurosurgery, University of Chicago Medicine, Chicago, IL 60637, USA; iawad@uchicago.edu; 7 Department of Radiology, University of Chicago, Chicago, IL 60637, USA; tjcarroll@uchicago.edu

**Keywords:** intracranial aneurysm, magnetic resonance imaging, microbleed, quantitative susceptibility mapping, sentinel headache, subarachnoid hemorrhage, vessel wall imaging

## Abstract

Background: MR-quantitative susceptibility mapping (QSM) can identify microbleeds (MBs) in intracranial aneurysm (IA) wall associated with sentinel headache (SH) preceding subarachnoid hemorrhage. However, its use is limited, due to associated skull base bonny and air artifact. MR-vessel wall imaging (VWI) is not limited by such artifact and therefore could be an alternative to QSM. The purpose of this study was to investigate the correlation between QSM and VWI in detecting MBs and to help develop a diagnostic strategy for SH. Methods: We performed a prospective study of subjects with one or more unruptured IAs in our hospital. All subjects underwent evaluation using 3T-MRI for MR angiography (MRA), QSM, and pre- and post-contrast VWI of the IAs. Presence/absence of MBs detected by QSM was correlated with aneurysm wall enhancement (AWE) on VWI. Results: A total of 40 subjects harboring 51 unruptured IAs were enrolled in the study. MBs evident on the QSM sequence was detected in 12 (23.5%) IAs of 11 subjects. All these subjects had a history of severe headache suggestive of SH. AWE was detected in 22 (43.1%) IAs. Using positive QSM as a surrogate for MBs, the sensitivity, specificity, positive predictive value, and negative predictive value of AWE on VWI for detecting MBs were 91.7%, 71.8%, 50%, and 96.6%, respectively. Conclusions: Positive QSM findings strongly suggested the presence of MBs with SH, whereas, the lack of AWE on VWI can rule it out with a probability of 96.6%. If proven in a larger cohort, combining QSM and VWI could be an adjunctive tool to help diagnose SH, especially in cases with negative or non-diagnostic CT and lumbar puncture.

## 1. Introduction

Sentinel headache (SH) is a sudden, intense, and persistent headache that precedes spontaneous subarachnoid hemorrhage (SAH) by days or weeks [1]. Earlier complaint of SH was elucidated in 4–43% of subjects with later presentation of SAH [2]. Possible explanations of SH include minor blood leakage from the intracranial aneurysm (IA) into the subarachnoid space [3], minor bleeding in IA walls [4], structural change of the aneurysm, and local vasospastic reaction triggering ischemia [5]. Given a wide spectrum of potential causes of severe headache, especially in the emergency department, it is important to identify objectively subjects with unruptured IAs presenting with SH who have aneurysmal microbleeds (MBs), as it is an eminent biomarker of later presentation of SAH.

It remains problematic if patients harboring IAs presenting with severe headache suggestive of SH have no evidence of visible SAH in routine imaging head computed tomography (CT). Guidelines for the management of aneurysmal SAH [6] state that acute diagnostic work-up should include non-contrast head CT, which, if non-diagnostic, should be followed by lumbar puncture (LP). However, a negative LP could not eliminate the presence of MBs on IAs associated with SH. Furthermore, traumatic taps are not uncommon, occurring in an estimated 10% of all LPs in the emergency department [7,8]. Therefore, having an objective test that could detect small bleeds or cerebral MBs could assist or replace the need for LP, in patients harboring IAs without evidence of SAH on the CT scan. 

Current medical imaging techniques enable detailed visualization of human vasculature by using CT, MRI, and angiography. Recently, imaging studies demonstrated the novel algorithm for semi-automatic reconstruction of the branches of the human aortic arch for blood perfusion analysis, for further use in computational fluid dynamic [9,10]. However, its accuracy needs to be improved, as further manual corrections are still needed [9,10]. Therefore, clinicians are motivated to use different techniques of medical image processing to improve the quality of assessment. 

Quantitative susceptibility mapping (QSM) is a MRI technique that provides a quantitative measure of tissue magnetic susceptibility using gradient–echo phase data [11]. Susceptibility weighted images generate contrast based on magnitude and filtered phase images, whereas QSM enables quantitative investigation of local tissue susceptibility, also allows distinguishing calcification which is hypo-intense, due to its diamagnetic properties from the hyperintense paramagnetic product of hemorrhage [12]. QSM directly visualized susceptibility changes within the blood clot and acute/chronic hemorrhage can be differentiated by QSM in vitro [13]. In vivo assessment of vascular permeability and iron deposition on MRI-QSM can serve as objective and quantifiable biomarkers of disease activity in cerebral cavernous malformations [14]. A recent study revealed that QSM could detect slight hemorrhages into the carotid plaque and may therefore be beneficial to evaluate the vulnerability of atherosclerotic plaques [15]. These studies suggested that QSM is reliable to assess the presence of MBs. 

Recently, our group reported that QSM could be used to detect MBs in the aneurysm walls associated with SH with a high sensitivity and specificity [16,17]. However, it was frequently difficult to completely visualize IAs, especially for those which were located close to skull base and air cells, because of skull base bone and air artifacts [17]. On the other hand, MR-vessel wall imaging (VWI) enables the artifact-free visualization of the thickened aneurysm wall with atherosclerosis, inflammation, and neovascularization as aneurysm wall enhancement (AWE), which might be a novel biomarker of IA instability [18,19]. The purpose of this study was to investigate the correlation between the QSM and AWE, and to assess whether this type of imaging could complement the conventional assessment of CT and LP, especially when they are negative or non-diagnostic, during the work-up of patients with IAs presenting with severe headache. Finally, we propose a strategy of management for IAs with SH.

## 2. Methods

### 2.1. Ethical Approval

This study was approved by the institutional review board (IRB) at the University of Iowa Hospitals and Clinics (local IRB No. 201811813). All patients provided written consent before enrollment in the study. 

### 2.2. Patients and Study Design

Subjects with an initial diagnosis of one or more unruptured IAs between November 2017 and June 2019 were prospectively recruited from the emergency department or the neurosurgery clinic. Patients who could not undergo MR imaging or could not use contrast material because of renal dysfunction were excluded. The diagnosis of unruptured IA was based on clinical presentation along with negative CT scan or MRI including fluid-attenuated inversion recovery for SAH, and a negative LP. SH was defined as a sudden onset, extremely severe, or worst headache with persistence within 2 weeks of presentation. These headaches were defined by the patient as different from any prior history of chronic headaches. 

We assigned patients a diagnosis of positive or negative SH based on the criteria above, and then subjected them to MRI imaging, to assess the utility of QSM and AWE in the setting of SH. For correlation between QSM and SH, subjects who had at least one IA with MBs were defined as positive QSM subjects. Subjects who had IAs with no detected MBs were defined as negative QSM subjects. We then assessed the sensitivity and specificity of AWE compared with QSM. We considered treatments for IAs based on guidelines for the management of patients with unruptured IAs [20], as well as patients’ preferences. Furthermore, aneurysm wall tissues were obtained from the subjects in whom partial resection of the wall of the fundus of the aneurysm was necessary to expose its neck during a microsurgical clipping. 

### 2.3. MR Acquisition

All patients underwent 3T-MRI (Siemens Skyra, Munich, Germany). The protocol for MRI included T1-weighted imaging, T2-weighted imaging, fluid attenuated inversion recovery, 3D time-of-flight (TOF) MR angiography (MRA), MRA with Gadolinium-based contrast agents (GBCA) gadobutrol (Gadvist, Bayer Pharmaceuticals, Whippany, NJ, USA), MRI-QSM and MR-VWI. MRI-QSM images were obtained using the following protocol: repetition time (TR), 61 ms; echo time (TE), 7–56 ms; flip angle, 17; pixel bandwidth, 260 Hz/pixel; field of view (FOV), 110.08 × 110.08 mm; matrix size, 256 × 256 × 64; voxel size, 0.43 × 0.43 × 1.50; slice thickness, 1.5 mm; frequency, 416; phase, 320; number of averages, 1. MRI-QSM images were generated offline using the STI Suite version 3.0 provided by University of California, Berkeley. Those for the VWI were as follows: TR, 900 ms; TE, 15 ms; flip angle, 120; pixel bandwidth, 445 Hz/pixel; FOV, 180.0 × 180.0 mm; matrix size, 320 × 320 × 64; voxel size, 0.56 × 0.56 × 1.0; slice thickness, 1.0 mm; echo train length, 52. GBCA gadobutrol was administered intravenously (0.1 mmol/kg), and the post-contract MR-VWI scan was performed 5 min after gadolinium infusion. 

### 2.4. Image Analysis and Detection of the MBs and AWE 

Two blinded adjudicators (D.I. and M.Z.), who are experienced vascular neurosurgeons, separately evaluated both the QSM and VWI images. In case of disagreement between two adjudicators, a consensus was found by discussion. The presence of MBs was evaluated by overlapping MRA images of the IA with QSM images using 3D slicer, an open-source software platform [21]. Segmentation for MBs was performed using pixel-labeling methods with the optimal susceptibility threshold of 0.1 parts per million [22]. The regions with a high susceptibility in QSM images were strictly distinguished from other cerebral vasculature. 

The contrast ratio between aneurysmal wall enhancement and pituitary stalk was used to define AWE, as described elsewhere [23,24]. The volume of interest with the highest signal intensity (SI) in the aneurysmal wall and the pituitary stalk on post-contrast imaging were defined. A region of interest of the aneurysm wall and pituitary stalk were drawn at the level of the maximum aneurysm diameter and in the whole pituitary stalk, respectively. Additionally, the averaged SI of the aneurysm circumferential wall (SIwall) and pituitary stalk (SIstalk) in each of the volumes of interest was measured using an open-source medical image viewer (Horos; https://horosproject.org). The positive AWE was defined as SIwall/SIstalk >0.39, based on the cutoff value between stable and unstable unruptured IAs demonstrated previously [24]. 

### 2.5. Statistical Analyses 

All statistical analyses were performed using JMP Pro version 14.0 (SAS Institute, Cary, NC, USA). Values are presented as the mean ± SD. Categorical variables were compared by the Fisher exact probability test. Continuous variables with normal distributions were analyzed by the Student’s *t* test and those with non-normal distributions were analyzed by the Mann–Whitney *U* test. The relationship between the presence of MBs and AWE was analyzed using a chi-square test. Interrater agreement for the detection of MBs and AWE was assessed by calculating the κ coefficient. Significance was defined as a *p*-value of less than 0.05. 

## 3. Results

### 3.1. Patient Demographics and Characteristics of IA

**Figure 1:**
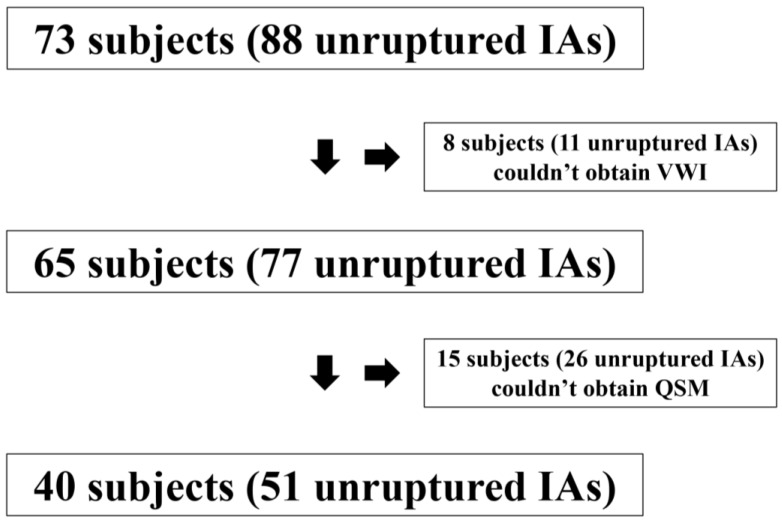
Flow chart—Summary of inclusion and exclusion for the patients.

Seventy-three consecutive patients with 88 unruptured IAs were consented for study. Eight patients with eleven unruptured IAs were excluded according to the exclusion criteria. Twenty-six of 77 IAs (33.8%) could not be visualized completely on QSM because of the skull base bone and air artifacts. Of these, 9 aneurysms (34.6%) were located in the supraclinoid segment of internal carotid artery, 5 (19.2%) in the cavernous segment of internal carotid artery, 5 (19.2%) in the anterior communicating artery, 2 (7.7%) in the middle cerebral artery, 4 (15.4%) in the trunk of basilar artery, and 1 (3.9%) in the union of vertebral arteries, respectively. The size of IAs which were not completely visualized in QSM due to the artifacts was significantly larger than those which could be analyzed in QSM (10.4 mm versus 7.3 mm, *p* = 0.03). These aneurysms were excluded as we intended to compare QSM with AWE. A total of 40 patients with 51 unruptured IAs were investigated (Figure 1). 

The average age was 62.4 ± 11.8, and 44 (86.3%) were female. All patients tolerated the imaging procedures without complications. Ten aneurysms (19.6%) were located in the internal carotid artery, 9 (17.6%) in the anterior cerebral artery, 16 (31.4%) in the middle cerebral artery, and 16 (31.4%) in the posterior circulation. The mean IA diameter was 7.3 ± 5.0 mm (range: 3.0–24.8 mm). Forty-five (88.2%) were saccular aneurysms, whereas 6 (11.8%) were fusiform. Seven IAs were treated by microsurgical clipping, 27 by endovascular procedures, and 17 were observed. Subjects’ demographics, IA characteristics, and IA features are summarized in Table 1.

### 3.2. Performance of QSM in Detecting SH

MBs were detected on QSM in 23.5% (12/51) of all IAs. MBs on QSM were significantly associated with increased size of the aneurysms (odds ratio (OR) = 1.19, 95% confidence interval (CI) = (1.05–1.41), *p* < 0.01). All MBs were located on the interface of each aneurysm with the brain parenchyma. The relationship between the presence of MBs on QSM and the presence of severe headache suggestive of SH is summarized in Table 2. 

**Figure 2:**
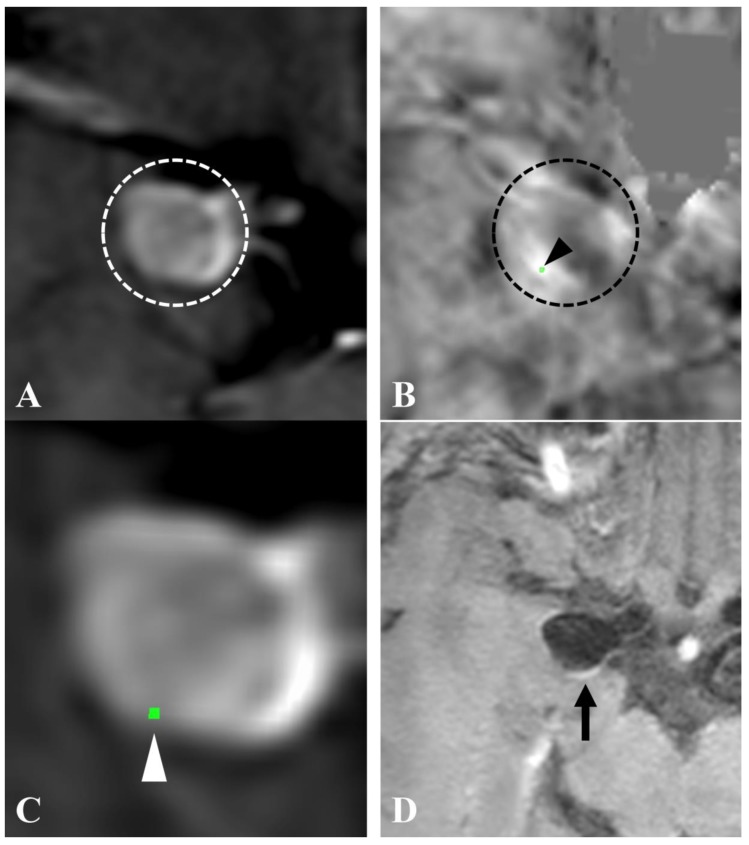
QSM and vessel wall imaging (VWI) of an intracranial aneurysm with SH. A subject presented to our neurosurgical clinic with sudden onset of a worst headache. There was no evidence of subarachnoid hemorrhage (SAH) in head CT and lumbar puncture. A right internal carotid artery–posterior communicating artery aneurysm was described in time-of-flight MR angiography (TOF-MRA) ((**A**), circle) and QSM ((**B**), circle). An area of high susceptibility, which was based on the threshold of 0.1 parts per million, was revealed ((**B**), arrowhead). The QSM image overlapped on TOF-MRA (**C**) indicated the area of high susceptibility at the interface of the aneurysmal wall (arrowhead). Postcontrast VWI (**D**) showed AWE in the aneurysmal wall (arrow).

Of the 40 subjects evaluated in the study, 11 subjects were diagnosed with SH by the criteria defined in the method section, and all had at least one IA with MBs on QSM. All patients with no history of SH had negative QSM (no MBs on QSM). A representative case is described in Figure 2. 

**Figure 3:**
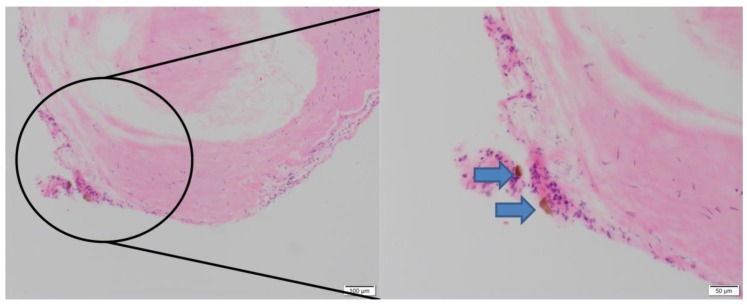
Immunohistochemical analysis of aneurysm dome tissue for hemosiderin. A subject with left terminal ICA aneurysm with presentation of SH and positive QSM shows presence of hemosiderin (blue arrows) on immunohistochemical hematoxylin and eosin staining of aneurysm wall tissue harvested during microsurgical clipping of aneurysm.

Aneurysm wall tissues were obtained from 3 IAs with positive QSM and 3 IAs with negative QSM. Immunohistochemical analysis was performed for hemosiderin. All 3 IAs with positive QSM showed hemosiderin, while those that were negative on QSM did not show hemosiderin (Figure 3). 

### 3.3. Performance of VWI Against SH

AWE was positive in 43.1% (22/51) of all IAs. Positive AWE on VWI was defined as SIwall/SIstalk >0.39 as described in the method section, based on previous published reports [24]. Eight IAs were enhanced partially and 14 were circumferentially. The mean SIwall/SIstalk of IAs with positive QSM was significantly higher than those of IAs with negative QSM (0.58 ± 0.27, 0.36 ± 0.13, respectively, *p* < 0.01). The relationship between detecting MBs on QSM and AWE is summarized in Table 3. 

The location of MBs and AWE were overlapped in 72.7% (8/11) of IAs with positive QSM as well as AWE. AWE was positive in all but one aneurysm with MBs. Positive QSM findings were significantly associated with the presence of MBs (*p* < 0.01). The sensitivity, specificity, positive predictive value (PPV), and negative predictive value (NPV) of AWE for detecting aneurysms with MBs (compared with QSM as gold standard) were: 91.7%, 71.8%, 50%, and 96.6%, respectively. Interrater agreement with respect to MBs detected on QSM and AWE was significantly high (MBs; 0.94, 95% CI = (0.91–0.99), *p* < 0.01, AWE; 0.96, 95% CI = (0.95–1.00), *p* < 0.01, respectively). 

## 4. Discussion

In this study, we have shown that QSM detected MBs in all patients with clinical SH, and the MBs were consistent with immunohistochemical analysis of aneurysm wall issue. Consequently, any unruptured IAs in patients suspected of suffering from SH, i.e., the presence of MBs on QSM, warrants urgent surgical/endovascular treatment. On the other hand, negative QSM and/or no AWE on VWI suggest strongly that the unruptured IA in question has no MBs and therefore could be observed. However, one third of IAs were not visualized completely with QSM because of the skull base bone and air artifacts, and thus were not analyzed for MBs. IAs with relatively larger dome and those located close to skull base and air cells tend to be affected by such artifacts. In these situations, we have found AWE to be useful due to the high NPV, as the lack of AWE on VWI can rule out the presence of MBs in IAs with a probability of 96.6%. Thus, IAs that could not be evaluated by QSM and have negative AWE can be considered stable and be observed over time. Our suggested pragmatic management strategy for IA with SH is described in Table 4. 

LP is routinely indicated in patients with severe headache suggestive of SH or SAH to detect minor bleeding from IAs, if no apparent SAH could be visualized on CT. However, a recent study reviewed 1286 LPs performed to rule out SAH in patients with negative head CT, and concluded that it did not identify any case of aneurysmal SAH, but was associated with serious complications, a significant false positive rate, and extended length of stay in the emergency department [25]. Considering the high incidence of non-diagnostic LP, QSM and VWI could be adjunctive tools to the assessment of LP for unruptured IAs when SH is suspected and there is no evidence of SAH on CT. In this study, since all the LPs were negative (inclusion criteria), we would have left 11/40 (27.5%) patients untreated due to missed MBs. 

In our cohort, 5 subjects who reported recent history (within 2 weeks) of severe headache suggestive of SH harbored 6 IAs. These subjects did not seek immediate care. However, the persistent new and interactable headache made them finally seek medical attention. These subjects had positive QSM. This suggests that QSM can detect recent and remote MBs. This is similar to published reports of the ability of QSM for detecting recurrent and additional hemorrhage or growth during the follow-up of cerebral cavernous malformations, after the initial presentation of recent bleed [26]. Based on that, QSM has been proposed as a biomarker for cerebral MBs [11]. As mentioned above, positive QSM findings was significantly associated with the size of IAs. Therefore, we proposed that subjects with IAs with MBs identified on QSM, and with no current suspicion of SH, may have had chronic MBs (asymptomatic leak) with aneurysmal growth. However, we did not find MBs outside the setting of SH in our cohort. Thus, we need more investigation to answer whether a patient harboring an IA with MBs on QSM without a history of SH has to be treated. 

AWE might be a novel biomarker of IA instability [18,19], since it is associated with inflammatory and/or atherosclerotic reaction along with thickening of the aneurysm walls [18,27]. Unstable unruptured IAs demonstrated AWE more frequently than stable ones [28,29,30]. Additionally, the pattern of AWE is significantly associated with the morphological changes observed in IAs, such as aneurysmal sac expansion or daughter sac formation [31]. It remains controversial if AWE could serve as potential imaging modality capable of identifying unstable IAs [32,33], as 20–40% of stable unruptured IAs may also demonstrate the AWE [19,31,34]. In our study, AWE did not have a high PPV for MBs associated with SH, but lack of AWE had a high NPV. A recent meta-analysis revealed that the sensitivity of the AWE for identifying unstable IAs was high (95%) [35]. Additionally, the NPV was also high (96%), suggesting that the absence of the AWE is strongly associated with IA stability [35]. It is also important to note that a sizeable proportion of IAs with AWE are stable, reflected by a PPV = 56% for instability [35]. This study suggested that the IAs with AWE are diverse in their stability. The observed high NPV for IAs with negative AWE was consistent with our results. 

### Limitations

Our current study has a small sample size and therefore a larger cohort is needed to confirm these findings. Based on the high percentage of non-diagnostic LP and the clinically unpredictable course of SH, findings from LP and non-contrast head CT to identify the presence or absence of MBs associated with SH are not reliable in the clinical setting. On the other hand, phantom studies confirmed without any doubt that positive QSM is an accurate presentation of non-hem iron and, therefore, hemosiderin [36]. The proposed management paradigm should not replace the clinical judgment but should be used as additional information when making a decision on whether to treat or not. 

The major limitation of the study is the categorization of clinical SH, which remains a subjectively defined entity. Given the several confirmatory studies outlined above, we have accepted in our study that a positive QSM is a biomarker for MBs. 

## 5. Conclusions

Positive QSM findings strongly suggested the presence of MBs with SH, whereas the lack of AWE on VWI can rule it out with a probability of 96.6%. The combinational assessment of QSM and VWI can be used reliably and the proposed strategy of management could be helpful to manage patients with unruptured IAs presenting with headaches. QSM and VWI have high sensitivity, specificity, and interrater agreement. If proven in a larger cohort, this combined assessment could be an adjunctive tool to the evaluation for patients with SH accompanied by negative or non-diagnostic CT and may potentially alleviate the need for LP.

## Figures and Tables

**Table 1:** Patient demographics and intracranial aneurysm (IA) characteristics.

Characteristics	Value
**Patients** Age ± SD (range), years Female Sex, *n* (%) SH, *n* (%) **Aneurysms** ICA, *n* (%)	62.4 ± 11.8 (43–85) 44 (86.3%) 11 (27.5%) 10 (19.6%)
ACA, *n* (%) MCA, *n* (%) Posterior circulation, *n* (%) Maximum diameter, mean ± SD (range) (mm) Saccular, *n* (%) Microbleeds, *n* (%) AWE, *n* (%) **Treatments** Microsurgical clipping, *n* (%) Endovascular procedure, *n* (%) Observation, *n* (%)	9 (17.6%) 16 (31.4%) 16 (31.4%) 7.3 ± 5.0 (3.0–24.8) 45 (88.2%) 12 (23.5%) 22 (43.1%) 7 (13.7%) 27 (52.9%) 17 (33.3%)

Abbreviations: ACA = anterior cerebral artery; AWE = aneurysm wall enhancement; IA = intracranial aneurysm; ICA = internal carotid artery; MCA = middle cerebral artery; SD = standard deviation; SH = sentinel headache.

**Table 2:** Relationship between microbleed detected by quantitative susceptibility mapping (QSM) and presentation of severe headache suggestive of SH.

		Subjects with Presentation of Severe Headache Suggestive of SH	
		Positive	Negative
**Subjects with**	Positive QSM	11	0
	Negative QSM	0	29

Abbreviations: QSM = quantitative susceptibility mapping; SH = sentinel headache.

**Table 3:** Relationship between the visualization of microbleeds detected by QSM and aneurysm wall enhancement.

		Microbleeds Detected by QSM	
		Positive	Negative
**AWE**	Positive	11	11
	Negative	1	28

Abbreviations: AWE = aneurysm wall enhancement; QSM = quantitative susceptibility mapping.

**Table 4:** Recommendation of surgical treatment for intracranial aneurysm with sentinel headache.

		QSM		
		Available		Unavailable
		Positive	Negative	
**AWE**	Positive	Strongly recommended	Might be considered	Might be considered
	Negative	Strongly recommended	Conservative	Conservative

Abbreviations: AWE = aneurysm wall enhancement; QSM = quantitative susceptibility mapping.
